# The Serotonin 4 Receptor Subtype: A Target of Particular Interest, Especially for Brain Disorders

**DOI:** 10.3390/ijms25105245

**Published:** 2024-05-11

**Authors:** Véronique Sgambato

**Affiliations:** 1 Institut des Sciences Cognitives Marc Jeannerod (ISCMJ), Unité Mixte de Recherche 5229 du Centre National de la Recherche Scientifique (CNRS), 69675 Bron, France; veronique.sgambato@inserm.fr; Tel.: +33-4379-11249; 2 UFR Biosciences, Université de Lyon 1, 69100 Villeurbanne, France

**Keywords:** serotonin, 5-HT_4_ receptor, depression, anxiety, cognitive decline, movement disorders, eating disorders, constipation

## Abstract

In recent years, particular attention has been paid to the serotonin 4 receptor, which is well expressed in the brain, but also peripherally in various organs. The cerebral distribution of this receptor is well conserved across species, with high densities in the basal ganglia, where they are expressed by GABAergic neurons. The 5-HT_4_ receptor is also present in the cerebral cortex, hippocampus, and amygdala, where they are carried by glutamatergic or cholinergic neurons. Outside the central nervous system, the 5-HT_4_ receptor is notably expressed in the gastrointestinal tract. The wide distribution of the 5-HT_4_ receptor undoubtedly contributes to its involvement in a plethora of functions. In addition, the modulation of this receptor influences the release of serotonin, but also the release of other neurotransmitters such as acetylcholine and dopamine. This is a considerable asset, as the modulation of the 5-HT_4_ receptor can therefore play a direct or indirect beneficial role in various disorders. One of the main advantages of this receptor is that it mediates a much faster antidepressant and anxiolytic action than classical selective serotonin reuptake inhibitors. Another major benefit of the 5-HT_4_ receptor is that its activation enhances cognitive performance, probably via the release of acetylcholine. The expression of the 5-HT_4_ receptor is also altered in various eating disorders, and its activation by the 5-HT_4_ agonist negatively regulates food intake. Additionally, although the cerebral expression of this receptor is modified in certain movement-related disorders, it is still yet to be determined whether this receptor plays a key role in their pathophysiology. Finally, there is no longer any need to demonstrate the value of 5-HT_4_ receptor agonists in the pharmacological management of gastrointestinal disorders.

## 1. Introduction

Serotonin (5-HT, 5-hydroxytryptamine) is a monoamine synthesized from an essential amino acid, L-tryptophan. L-tryptophan can cross the blood–brain barrier and be converted by the enzyme tryptophan hydroxylase 2 (TPH2) into L-5-hydroxytryptophan. The latter is then converted to 5-HT after decarboxylation by aromatic amino acid decarboxylase (AADC). TPH2 is specific to serotonergic neurons in the central nervous system and is responsible for 5-HT synthesis in the brain [1,2], while AADC is localized in many cell types. In the brain, the majority of the cell bodies of serotonin-containing neurons are located in the brain stem and grouped into nine nuclei (named B1 to B9) which are, in turn, divided into the rostral or superior group and the caudal or inferior group [3,4]. The rostral group is located in the pons and midbrain and consists of both the dorsal nucleus (DRN) and medial nucleus (MRN) of the raphe [5,6]. The caudal group is located in the medulla and consists of nuclei projecting mainly to the spinal cord [5]. Although, in many brain territories, projections from serotonergic neurons originate from both the DRN and MRN, in some regions, 5-HT innervation originates solely or preferentially from the DRN or MRN. This is the case, for example, of the hippocampus, the dorsal part of which receives serotonergic innervation from the MRN [7]. On the other hand, the 5-HT fibers of the striatum, globus pallidus, lateral septum, and amygdala, including the prefrontal cortex, come from the DRN [8]. Along with the MRN, the DRN innervates the ventral part of the hippocampus and the prefrontal cortex [8] and certain nuclei of the thalamus [7,9,10]. There are also dense serotonergic projections between the DRN and MRN [11]. The central nervous system therefore contains a small number of 5-HT neurons grouped together in nuclei, but these neurons release serotonin diffusely throughout the brain at chemical synapses or by volume transmission [4,12]. Serotonin can therefore exert a modulating action on a wide variety of neuronal circuits and therefore functions [13,14]. The action of serotonin is complicated by the identification of seven families of serotonin receptor subtypes (5-HT_1_ to 5-HT_7_ receptors) in the brain [15]. Six of these are metabotropic G-protein-coupled receptors and one (the 5-HT_3_ receptor) is a ligand-gated ion channel (ionotropic receptor) [16]. This leads to the existence of eighteen 5-HT receptor subtypes plus a number of isoforms resulting from alternative mRNA splicing [17]. While all 5-HT receptor subtypes have post-synaptic expression on target cells, some subtypes, such as 5-HT_1A_, 5-HT_1B/D_, and 5-HT_2B_, are also localized pre-synaptically on the 5-HT neurons themselves (autoreceptors). Finally, 5-HT receptors also differ in their brain expression profiles in the brain [18] and in their interactions with other neurotransmitter systems.

In recent years, particular attention has been focused on the serotonin 4 receptor subtype [19,20,21], originally identified and cloned in rodents [22,23]. This receptor is positively coupled to adenylate cyclase via a Gs-type G protein leading to the canonical activation of the cAMP/PKA pathway [24]. But activation of the 5-HT_4_ receptor can also lead to the activation of the ERK signaling pathway independently of G proteins [25]. In addition to the fact that the 5-HT_4_ receptor has a constitutive activity, which is expressed at low receptor levels, another important feature of the receptor is that it can establish homodimers or heterodimers with several other proteins. These dimers functionally impact the signaling pathways on which they act [26]. In the brain, the 5-HT_4_ receptor is mainly located on neurons and has a dual somatodendritic and axonal localization [27]. Its cerebral distribution is highly conserved across species [27,28] with the highest densities in basal ganglia, particularly in the striatum [29,30] and the substantia nigra [31]. The 5-HT_4_ receptor is expressed by GABAergic neurons, as neurotoxic lesions of rodent basal ganglia components lead to dramatic decreases in 5-HT_4_ receptor binding in these regions [27,32]. The post-synaptic expression of the 5-HT_4_ receptor seems independent of dopaminergic neurons, as a dopaminergic lesion induced by intranigral injection of the 6-hydroxydopamine in rodents does not induce decreases in its expression in the striatum or the substantia nigra [27,32]. Similarly, the lesion of serotonergic neurons by intra-raphe injection of the 5,7-dihydroxytryptamine upregulates the 5-HT_4_ receptor binding in the basal ganglia and hippocampal regions [32]. The 5-HT_4_ receptor is also highly expressed in the cerebral cortex, hippocampus, and amygdala, where it may be carried by glutamatergic or cholinergic neurons, depending on the region [33,34]. The distribution of this receptor has also been studied in the post-mortem human brain by in situ hybridization and ligand binding studies and has been found to be high in basal ganglia regions and moderate to low in hippocampal formation and the cortical mantle [28]. Substantial evidence from preclinical and clinical studies have indicated an inverse relationship between the central 5-HT tonus and the density of the 5-HT_4_ receptor. In animals, 5-HT depletion or 5-HT boosting by the use of antidepressants, or again serotonin transporter gene manipulation, all lead to alterations of the 5-HT_4_ receptor binding in the brain [35,36,37]. In humans, the polymorphism of the serotonin transporter also results in different 5-HT levels and to different expression levels of the 5-HT_4_ receptor [38]. And as it has been demonstrated that the density of the 5-HT_4_ receptor adapts to the variations in brain extracellular 5-HT levels induced by antidepressant treatment, the central 5-HT_4_ receptor binding is now used as an in vivo biomarker of serotonergic tonus [39]. Outside the central nervous system, the 5-HT_4_ receptor is known to be also expressed in the gastrointestinal tract, the cardiovascular system, the bladder, and the adrenal glands [40].

The 5-HT_4_ receptor is involved in several functions. It mediates a positive feedback effect on the 5-HT neurons themselves. Indeed, the 5-HT_4_ receptor in the prefrontal cortex has a positive influence on the rate of discharge of 5-HT neurons in the raphe and increases the probability of action potential emissions via the positive feedback loop from the prefrontal cortex to the dorsal raphe [41,42]. The administration of 5-HT_4_ agonists also activates raphe 5-HT neurons [41,43]. It also releases 5-HT in the hippocampus [44]. The action of 5-HT_4_ receptors in the hippocampus plays an important role in the regulation of synaptic plasticity and related memory mechanisms [45]. Apart from the serotonergic system, the modulation of the 5-HT_4_ receptor has effects on the homeostasis of other neurotransmitter systems such as acetylcholine or dopamine [46]. The stimulation of the 5-HT_4_ receptor facilitates the release of acetylcholine in the frontal cortex of awake rats [47]. Similarly, the 5-HT_4_ receptor has been shown to facilitate dopamine release in the rat striatum both in vitro and in vivo [48]. The fact that modulation of the 5-HT_4_ receptor can affect the release of different neurotransmitters highlights the importance of this receptor as a potential therapeutic player in a variety of pathological conditions.

### 1.1. Mood and Anxiety Disorders

One of the major interests of this receptor is its potential involvement in mood and anxiety disorders (Table 1). It has already been shown that the 5-HT_4_ receptor expression is altered in various rodent models of depression. Some studies have reported a decrease in 5-HT_4_ receptor expression [35,49], while others have observed an increase [50]. It is highly probable that these divergent results are due to the use of different animal models of depression. On the contrary, similar results were obtained by studies modulating 5-HT_4_ receptor activation. Drugs that activate the 5-HT_4_ receptor play a fast-acting beneficial role on behavioral markers of depression or anxiety [51]. Electrophysiological, molecular, morphological, and behavioral changes that have previously been specifically linked to beneficial long-term treatment with selective serotonin reuptake inhibitors are already present after only 3 days of treatment when using 5-HT_4_ agonists in rodents [52]. The lack of 5-HT_4_ receptor modulates mood-like disorders [53] and further impacts the response to antidepressants in rodents [54]. Overexpression of the 5-HT_4_ receptor in the medial prefrontal cortex leads to antidepressant effects [55], whereas its functional ablation throughout the brain [54] or only in the hippocampus [53] causes anxiety and mood disorders. The pharmacological modulation of the 5-HT_4_ receptor located in the lateral habenula also produces antidepressant effects, via changes in monoamine levels in the prefrontal cortex and hippocampus in parkinsonian rats [56]. Yet, links have been shown between the antidepressant action of 5-HT_4_ receptor agonists and mechanisms of synaptic plasticity or gene regulation in the hippocampus. For example, the administration of 5-HT_4_ receptor agonists leads to the upregulation of mRNAs encoding BDNF (brain-derived neurotrophic factor) in the hippocampus. This increase results from activation of the CREB (cAMP-response element-binding protein) signaling pathway, which stimulates the expression of various neurotrophic factors in the hippocampus, including BDNF [57]. Furthermore, it has been shown that the stimulation of the 5-HT_4_ receptor drives neurogenesis in the hippocampus, while the lack of or the antagonism toward the 5-HT_4_ receptor reduces these effects [58,59,60,61]. Furthermore, it has been shown that the activation of the 5-HT_4_ receptor boosts the formation and the function of dendritic spines [62,63] and more generally acts as a unique modulator of synaptic plasticity mechanisms in the hippocampus [64].

In support of these preclinical data, several human studies have also shown that expression levels of the 5-HT_4_ receptor are altered in mood disorders and/or depression (Table 1). Autopsy results of brain tissue from antidepressant-free, violent suicide victims have shown a high density of the 5-HT_4_ receptor in the frontal cortex and caudate nucleus compared with control subjects [65]. In vivo PET (positron emission tomography) data have shown a lower cerebral 5-HT_4_ receptor binding in patients with major depressive disorder than in healthy subjects [66,67]. And concomitant anxiety in patients suffering from depression is also negatively associated with 5-HT_4_ receptor brain binding [68]. There is also an association between brain 5-HT_4_ receptor binding and reactivity to emotional faces in depressed and healthy individuals: the higher the concomitant anxiety, the lower the binding in the brain [69]. The global brain binding of this receptor has also been positively linked to high trait aggression in men [70]. Finally, a polymorphism in the 5-HT_4_ receptor in its C-terminal region has been linked to unipolar depression in humans [71], suggesting that genomic variations of this gene may confer susceptibility to mood disorders. A more recent study aimed to assess the association of the 5-HT_4_ receptor polymorphism (rs1345697) with depressive symptom improvement after 6 months of antidepressant treatment and found that there is an impact of this rs1345697 polymorphism on the improvement in depressive symptoms and remission of these symptoms after antidepressant treatment [72]. Taken together, these preclinical and clinical data tend to show that there is rather a drop in expression of the 5-HT_4_ receptor associated with mood and anxiety disorders, and that the use of agonists of this receptor could reduce them.

**Table 1:** List and main characteristics of preclinical and clinical studies involving the 5-HT_4_ receptor in mood and anxiety disorders.

Goal of the Study	Methods of 5-HT_4_ Receptor Investigation	Models	Main Results		Reference
5-HT_4_ receptor polymorphism	Polymorphism	Bipolar or depressed individuals	C-terminal region linked to mood disorders *		[71]
		Major depressed individuals	Genotype GG of rs1345697 polymorphism associated with lower remission rates		[72]
5-HT_4_ receptor expression	Binding assays	Violent suicide human victims	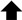	in frontal cortex *	[65]
			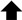	in caudate nucleus *	
	Autoradiography	Rat depression model	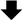	in the hippocampus	[35]
			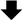	in hippocampus and basal ganglia under chronic paroxetine	
			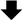	in hippocampus, hypothalamus, and lateral globus pallidus after 5-HT depletion	
		Mouse depression models	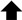	in ventral hippocampus	[50]
			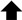	in caudal caudate putamen	
	Deletion	KO mouse	Attenuation of “dematuration” of hippocampal granule cells		[58]
			Anxiety and anhedonia induced and responses to antidepressant impacted		[54]
	qRT-PCR, Western blot	Rat depression models	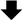	in hippocampus associated with anhedonia	[49]
	qRT-PCR	Casein kinase 2 KO mouse	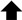	in prefrontal cortex associated with anti-depressant-like phenotype	[55]
	In situ hybridization	KO mouse	Fluoxetine-induced neurogenesis abolished		[61]
	Overexpression	Mouse	Anti-depressant-like phenotype		[55]
	PET imaging	Healthy individuals	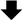	in striatum and limbic regions negatively correlated with number of first-degree relatives treated for major depression	[66]
			Positive correlation with high trait aggression, only in males		[70]
		Anxious and depressed individuals	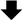	in global brain	[68]
		Depressed individuals	Negative correlation with concomitant anxiety *		[69]
			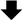	in global brain *	[67]
5-HT_4_ receptor modulation	5-HT_4_ receptor agonist	Rat depression models	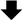	Depressive-like behavioral responses	[52]
		Rat	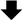	Depressive- and anxiety-like behavioral responses	[57]
		Rat model of Parkinson’s disease	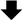	Depressive-like behavioral responses	[56]
		KO mouse	Antidepressant behavioral responses		[53]

* in comparison with healthy individuals. Up arrows indicate an increase, down arrows a decrease.

### 1.2. Cognitive Disorders

Another attraction of the 5-HT_4_ receptor subtype is that its activation can improve learning and memory (Table 2). Several preclinical experimental paradigms have evidenced such pro-cognitive properties [73,74]. Significant and reproducible improvements in a delayed matching to sample task accuracy are observed following an oral administration of a 5-HT_4_ agonist in both younger and older monkeys [73]. In rodents, activation of the 5-HT_4_ receptor leads to an increase in acetylcholine release, which counteracts memory deficits [21,46]. And it has been shown that the 5-HT_4_ receptor plays a specific and unique role in modulating the long-term potentiation of synapses in the hippocampus [75] and thus the memory mechanisms dependent on this structure [64]. It also promotes here the maturation of neuronal dendritic spines [63]. In humans, PET imaging studies have shown an inverse relationship between brain 5-HT_4_ receptor binding and memory performance in healthy subjects [76,77]. Even weaker brain binding is associated with memory dysfunction in untreated depressive patients compared with healthy individuals [67]. Finally, as in animals, stimulation of the 5-HT_4_ receptor by prucalopride improves cognitive performance in healthy subjects [78,79]. This beneficial effect is achieved by enhancing the resting-state functional connectivity between regions involved in cognitive networks while reducing that of the default mode network [80].

Stimulation of 5-HT_4_ receptors is also proving promising in various pathological situations. In a rat model of Alzheimer’s disease, the administration of a 5-HT_4_ receptor agonist improves cognitive performance via a reduction in apoptotic neurons and an improvement in synaptic function in the hippocampus [84]. In humans with early Alzheimer’s disease, a PET study elegantly showed that there is an increase in brain 5-HT_4_ receptor binding (parietal cortex, lateral prefrontal cortex, lateral temporal cortex, posterior cingulate gyrus, and hippocampus), and that this global cerebral upregulation is positively correlated with the cortical detection of beta-amyloid accumulation [81]. These preclinical and clinical data indicate that upregulation of the 5-HT_4_ receptor in the brain is beneficial, particularly in the hippocampus, where 5-HT_4_ receptor stimulation favors synaptic plasticity and memory mechanisms. This also suggests that increased expression of this receptor is a compensatory mechanism designed not only to counteract the accumulation of amyloid plaques [85], but also to promote acetylcholine release [47] and improve cognition.

Pro-cognitive effects are also mediated by 5-HT_4_ receptors in Parkinson’s neurodegenerative disease and may be underpinned by similar mechanisms. Indeed, the systemic administration of a 5-HT_4_ agonist improves the facilitation of contextual fear extinction, although without affecting hippocampal 5-HT_4_ receptors at the mRNA level, in the parkinsonian mice [83]. But another study on the parkinsonian rat has shown that lesioning the dopaminergic neurons of the median forebrain bundle produces cognitive deficits which are associated with a hippocampal increase in the receptor protein expression. Moreover, the local modulation of this receptor can counteract the memory deficits displayed by the animal, notably via increases in dopamine and serotonin in the hippocampus and the amygdala [82]. This suggests that, once again, the increased expression of the 5-HT_4_ receptor is a compensatory mechanism that stimulates the release of other neurotransmitters, thereby counteracting memory deficits.

Taken together, these data indicate that the stimulation of 5-HT_4_ receptors triggers a number of possible mechanisms (release of acetylcholine, serotonin, or dopamine; stimulation of maturation and synaptic plasticity in the hippocampus; induction of mechanisms able to counteract the accumulation of toxic aggregates and neuronal apoptosis), aimed at improving cognitive impairments. They also strengthen the view that 5-HT_4_ receptor agonists could be used in humans under different conditions: in subjects without a neurodegenerative pathology to improve memory deficits, and in elderly subjects affected by several evolutive diseases involving severe cognitive impairment [86].

### 1.3. Movement Disorders

Few studies have addressed the question of whether the 5-HT_4_ receptor subtype is in any way involved in movement disorders (Table 3). However, some animal models exist for inducing motor deficits or complications, in which either the impact of the pharmacological modulation of this receptor or its potential alteration of expression has been analyzed. In a rat model of morphine-driven stereotyped behavior and haloperidol-induced catalepsy, the systemic pharmacological blockade of the 5-HT_4_ receptor has no impact, suggesting that 5-HT_4_ receptor antagonism does not modulate motor behaviors related to the change in dopamine transmission [87]. Similarly, the turning behavior induced by amphetamine in unilateral dopamine-denervated rats is not affected after antagonizing the 5-HT_4_ receptor [88]. Divergent results were obtained in the parkinsonian mice. One study showed that the administration of 5-HT_4_ receptor agonists did not improve motor impairment [83]. On the contrary, a 7-day administration of prucalopride, a 5-HT_4_ agonist, induced a beneficial effect on motor deficits (bradykinesia, exploration, balance, and muscle strength) in the parkinsonian mice, by attenuating the striatal loss of dopamine [89]. Studies that have simply analyzed variations in receptor expression without using pharmacological ligands show divergent results. Changes in the striatal expression of the 5-HT_4_ receptor are not observed after dopaminergic lesion induction, nor after a chronic levodopa treatment, leading to abnormal involuntary movements (i.e., dyskinesias) in a rat model of Parkinson’s disease [90]. By contrast, using in vivo PET imaging and immunohistochemistry on post-mortem tissue, we found that the 5-HT_4_ receptor is upregulated in the striatum of parkinsonian macaques and rats, and further increased following chronic levodopa treatment, inducing dyskinesias in both animal species [91]. In humans, binding studies have not revealed any significant differences in 5-HT_4_ receptor expression in the putamen or the substantia nigra between control subjects and Parkinson’s sufferers, although there is a tendency for this receptor to increase in the putamen of patients [92,93]. By contrast, in Huntington’s patients, who exhibit involuntary movements, a marked putaminal decrease in the 5-HT_4_ receptor density is found [92]. However, a 3-week treatment with mosapride, a 5-HT_4_ receptor agonist, improved motor functions in a small number of Parkinsonian patients [94]. Taken together, these results show that the question of possible links between altered 5-HT_4_ receptor expression in the brain and movement disorders remains a largely open question. Further studies are sorely needed to elucidate whether or not this 5-HT_4_ receptor plays a role in the pathophysiology of movement disorders.

### 1.4. Food Disorders

Several studies, first in rodents and non-human primates, then in humans, have shown that the 5-HT_4_ receptor is associated with the control of food intake (Table 4). In normal or food-deprived mice, the local stimulation of the 5-HT_4_ receptor in the nucleus accumbens, a region involved in the reward system, inhibits food intake, an effect that is not obtained in 5-HT_4_-receptor-deficient mice [95]. The same group has also shown that deletion or overexpression of the 5-HT_4_ receptor in the medial prefrontal cortex induces overeating or hypophagia, respectively [96]. In obese rats, there is a higher level of the accumbal 5-HT_4_ receptor, whose stimulation with an agonist reduces eating [97]. In normal macaque monkeys, here again, the modulation of this receptor reduces or enhances the motivation for food intake, following systemic administration of a 5-HT_4_ receptor agonist or antagonist, respectively (personal communications). Similarly, in dopamine-lesioned macaques, the local injection of a 5-HT_4_ agonist into the ventral striatum (the rodent equivalent of the nucleus accumbens) triggers a reduced motivation for food intake (personal communications). In humans, a PET imaging study found that obesity is associated with the greater availability of this receptor in the nucleus accumbens and ventral pallidum of the brain reward circuit [98]. All these preclinical and clinical data point in the same direction and show that the high expression of 5-HT_4_ receptors in the nucleus accumbens is associated with a high body mass index, and that the stimulation of 5-HT_4_ receptors helps reduce the food intake.

### 1.5. Other Brain Disorders

Finally, one clinical study has reported an association between the methylation of the 5-HT_4_ receptor promoter and autism spectrum disorder [99]. Another has linked hippocampal structural changes in psychosis to the distribution of the 5-HT_4_ receptor [100], opening new avenues of research on this receptor. More recently, a study investigating the possible involvement of the serotonergic system in the pathophysiology of amyotrophic lateral sclerosis showed, using a transgenic mouse model, that systemic administration of a 5-HT_4_ receptor antagonist worsened the disease’s phenotype. It degraded motor function, without affecting disease progression. However, the 5-HT_4_ receptor antagonist also induced an increase in the expression of proteins forming abnormal aggregates, as well as an increase in the number of glial cells at the expense of neurons, in these mice [101], suggesting that using 5-HT_4_ receptors agonists might be of particular interest.

### 1.6. Gastrointestinal Disorders

Interestingly also, the 5-HT_4_ receptor agonists have been evaluated as prokinetics in the gastrointestinal tract (Table 5). The gavage of prucalopride causes faster whole gut transit and colonic motility and these effects are blocked by a 5-HT_4_ receptor antagonist and are not detected in 5-HT_4_ receptor knockout mice [102]. Sacral nerve electrical stimulation can improve defecation and accelerate the recovery of colonic transmission functions in a rat model of constipation, and these effects involve an upregulation of the 5-HT_4_ receptor in the colon [103]. The 5-HT_4_ receptor agonists prucalopride and tegaserod have both been approved by the FDA (Food and Drug Administration) and are used to treat various gastrointestinal-related conditions, including nausea, diarrhea, gastroparesis, constipation, and pain in irritable bowel syndrome [104]. In patients suffering from chronic idiopathic constipation [104], prucalopride is highly efficient, and other highly selective 5-HT_4_ receptor agonists are still in advanced development [105]. In other pathologies, such as Parkinson’s disease, 5-HT_4_ receptor agonists can also improve non-motor symptoms such as constipation. This has been demonstrated both preclinically and clinically. Rats with bilateral lesions of nigral dopaminergic neurons exhibit a reduced 5-HT_4_ receptor expression in the colonic muscular layer and manifest constipation, which is counteracted by a 5-HT_4_ receptor agonist (cisapride) [106]. In the MPTP (1-methyl-4-phenyl-1,2,3,6-tetrahydropyridine)-treated mice, a beneficial effect is obtained on the integrity of the intestinal barrier after intraperitoneal administration of prucalopride, the 5-HT_4_ receptor agonist, for 7 days [89]. Clinical studies have also tested the effects of the pharmacological stimulation of the 5-HT_4_ receptor on constipation in Parkinson’s disease (see [107]) (Table 5). It has been shown that administration of the 5-HT_4_ receptor agonist mosapride for 3 months improves the intestinal transit in patients without worsening their parkinsonian symptoms or causing serious side-effects [108]. Another study involving a small number of parkinsonian patients showed an increasing gastric motility during mosapride treatment [94]. These data are interesting insofar as constipation is a non-motor symptom that may precede the onset of motor symptoms in Parkinson’s disease. We could therefore imagine that the therapeutic targeting of the 5-HT_4_ receptor could be modulated according to the symptom (motor or non-motor), but also according to the stage of the pathology (early, advanced, or late).

## 2. Conclusions

All these studies, both preclinical and clinical, highlight this 5-HT_4_ receptor as a potential therapeutic target, especially for various brain disorders. While the benefits of activating this receptor have been well demonstrated at the preclinical level for certain conditions, such as depressive symptoms, memory deficits, food consumption, and constipation, there is still some way to go in terms of the interest in acting pharmacologically on this receptor in the field of movement disorders. In humans, the activation of this receptor to counteract certain disorders is promising but remains to be validated, since only gastrointestinal disorders can be improved so far by using compounds acting toward this receptor. Further studies are sorely needed to unravel all the mysteries surrounding this receptor, both in terms of its functions and its potential therapeutic use.

## Figures and Tables

**Table 2:** List and main characteristics of preclinical and clinical studies involving the 5-HT_4_ receptor in cognitive disorders.

Goal of the Study	Methods of 5-HT_4_ Receptor Investigation	Models	Main Results		Reference
5-HT_4_ receptor expression	PET imaging	Subjects with early Alzheimer’s disease	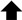	In global brain and positively correlated with beta-amyloid accumulation	[81]
		Healthy young adult individuals	Inverse relationship with memory performance		[76]
			Inverse relationship with positive and neutral word recall performance		[77]
		Depressed patients	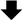	in global brain * associated with memory dysfunction	[67]
	Western blot	Rat model of Parkinson’s disease	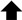	in dorsal hippocampus	[82]
5-HT_4_ receptor modulation	5-HT_4_ receptor agonist	Macaque young and old)	Learning improved		[73]
		Mouse	Memory improved		[74]
		Mouse model of scopolamine-induced deficit	Memory deficits counteracted		[46]
		Mouse model of Parkinson’s disease	Restored facilitation of contextual fear extinction		[83]
		Rat model of Parkinson’s disease	Working memory improved		[82]
		Rat model of Alzheimer’s disease	Memory deficits and associated mechanisms counteracted		[84]
		Healthy young adult individuals	Pro-cognitive effects induced		[78]
			Mnesic performance improved associated with increased hippocampal activity		[79]
	5-HT_4_ receptor agonist and antagonist	ePet1-cre; Ai32 mouse	Memory formation modulated		[75]

* in comparison with healthy individuals. Up arrows indicate an increase, down arrow indicates a decrease.

**Table 3:** List and main characteristics of preclinical and clinical studies involving the 5-HT_4_ receptor in movement disorders.

Goal of the Study	Methods of 5-HT_4_ Receptor Investigation	Models	Main Results		Reference
5-HT_4_ receptor expression	Binding assays	Patients affected by Huntington’s disease	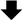	in putamen *	[92,93]
		Patients affected by Parkinson’s disease	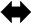	in putamen and nigra *	[92,93]
	In situ hybridization	Rat model of Parkinson’s disease	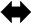	in striatum	[90]
	PET imaging immunohistochemistry		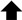	in motor striatum	[91]
5-HT_4_ receptor modulation	5-HT_4_ receptor agonist	Patients affected by Parkinson’s disease	Motor functions improved		[94]
		Mouse model of Parkinson’s disease	No motor improvement		[83]
			Motor deficits improved associated with rescued striatal dopamine levels		[89]
	5-HT_4_ receptor antagonist	Rat model of amphetamine-induced turning behavior	No impact		[88]

* in comparison with healthy individuals. Up arrow indicates an increase, down arrow a decrease. Horizontal arrows indicate no change.

**Table 4:** List and main characteristics of preclinical and clinical studies involving the 5-HT_4_ receptor in food disorders.

Goal of the Study	Methods of 5-HT_4_ Receptor Investigation	Models	Main Results		Reference
5-HT_4_ receptor expression	Autoradiography	Rat model of obesity	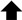	In nucleus accumbens	[97]
	PET imaging	Normal to high body mass index individuals	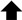	In nucleus accumbens, ventral pallidum, left hippocampus, and orbitofrontal cortex, associated with obesity	[98]
	Deletion	Mouse	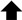	Food intake	[96]
	Overexpression	Mouse	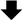	Food intake	[96]
	Food intake modulated	KO mouse	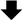	Food intake	[96]
5-HT_4_ receptor modulation	5-HT_4_ receptor agonist or antagonist	Mouse	Food intake modulated		[95]
		Macaque	Modulated food intake		personal communications
	5-HT_4_ receptor agonist	Macaque model of Parkinson’s disease	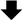	Food intake	personal communications

Up arrows indicate an increase, down arrows a decrease.

**Table 5:** List and main characteristics of preclinical and clinical studies involving the 5-HT_4_ receptor in gastrointestinal disorders.

Goal of the Study	Methods of 5-HT_4_ Receptor Investigation	Models	Main Results		Reference
5-HT_4_ receptor expression	ELISA, qRT-PCR, immunohistochemistry, Western blot	Rat model of constipation	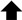	In colon after sacral nerve electrical stimulation	[103]
	Western blot	Rat model of Parkinson’s disease	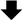	In colon, associated with constipation	[106]
5-HT_4_ receptor modulation	5-HT_4_ receptor agonist	Patients affected by Parkinson’s disease	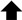	Intestinal transit	[108]
			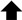	Gastric motility	[94]
		Rat model of Parkinson’s disease	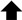	In colon	[106]
			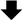	Constipation	
		Mouse model of Parkinson’s disease	Beneficial effect on intestinal barrier integrity		[89]
	5-HT_4_ receptor agonist and antagonist	Naïve or KO mouse	Modulated gut transit and colonic motility		[102]

Up arrows indicate an increase, down arrows a decrease.
